# Synthesis, Characterization, and Antibacterial Activity of Cross-Linked Chitosan-Glutaraldehyde

**DOI:** 10.3390/md11051534

**Published:** 2013-05-13

**Authors:** Bin Li, Chang-Lin Shan, Qing Zhou, Yuan Fang, Yang-Li Wang, Fei Xu, Li-Rong Han, Muhammad Ibrahim, Long-Biao Guo, Guan-Lin Xie, Guo-Chang Sun

**Affiliations:** 1 State Key Laboratory of Rice Biology, Institute of Biotechnology, Zhejiang University, Hangzhou 310058, China; E-Mails: libin0571@zju.edu.cn (B.L.); changlin_shan@163.com (C.-L.S.); zhou-0725-qing@163.com (Q.Z.); ibrahim@ciitsahiwal.edu.pk (M.I.); glxie@zju.edu.cn (G.-L.X.); 2 State Key Laboratory Breeding Base for Zhejiang Sustainable Plant Pest and Disease Control, Zhejiang Academy of Agricultural Sciences, Hangzhou 310021, China; E-Mail: wangylaa@yahoo.com.cn; 3 College of Chemistry and Life Sciences, Zhejiang Normal University, Jinhua 321004, China; E-Mail: Fy0579@zjnu.cn; 4 Institute of Digital Agriculture, Zhejiang Academy of Agricultural Sciences, Hangzhou 310021, China; E-Mail: fxu@zju.edu.cn; 5 Research and Development Center of Biorational Pesticides, Northwest A & F University, Yangling, Shaanxi 712100, China; 6 State Key Laboratory of Rice Biology, China National Rice Research Institute, Hangzhou 310006, China

**Keywords:** antibacterial activity, characterization, chitosan, cross-link, glutaraldehyde

## Abstract

This present study deals with synthesis, characterization and antibacterial activity of cross-linked chitosan-glutaraldehyde. Results from this study indicated that cross-linked chitosan-glutaraldehyde markedly inhibited the growth of antibiotic-resistant *Burkholderia cepacia* complex regardless of bacterial species and incubation time while bacterial growth was unaffected by solid chitosan. Furthermore, high temperature treated cross-linked chitosan-glutaraldehyde showed strong antibacterial activity against the selected strain 0901 although the inhibitory effects varied with different temperatures. In addition, physical-chemical and structural characterization revealed that the cross-linking of chitosan with glutaraldehyde resulted in a rougher surface morphology, a characteristic Fourier transform infrared (FTIR) band at 1559 cm^−^^1^, a specific X-ray diffraction peak centered at 2*θ* = 15°, a lower contents of carbon, hydrogen and nitrogen, and a higher stability of glucose units compared to chitosan based on scanning electron microscopic observation, FTIR spectra, X-ray diffraction pattern, as well as elemental and thermo gravimetric analysis. Overall, this study indicated that cross-linked chitosan-glutaraldehyde is promising to be developed as a new antibacterial drug.

## 1. Introduction

The *Burkholderia cepacia* complex (Bcc) is a collection of genetically distinct but phenotypically similar bacteria that have emerged as life-threatening pulmonary pathogens in immunocompromised patients, particularly individuals with cystic fibrosis (CF). Indeed, the Bcc currently comprises 17 different species and infection with Bcc often leads to a fast decline in lung function and a markedly increased mortality [1,2,3]. The number of infections caused by the Bcc is increasing in China [4,5,6], while some species such as *Burkholderia cenocepacia*, *Burkholderia contaminans*, *Burkholderia multivorans*, *Burkholderia seminalis*, *Burkholderia stabilis*, and *Burkholderia vietnamiensis* have been isolated from agricultural and hospital environments in our previous studies [6,7,8,9,10].

Treatment of CF infections is very difficult due to the intrinsic resistance of Bcc bacteria to most clinically useful antibiotics, while some isolates of Bcc even can utilize penicillin G as a sole carbon source for growth [6,11]. Thus, it becomes important to identify newer and improved antibacterial therapies for CF patients. Recently, chitosan, a natural nontoxic biopolymer derived by deacetylation of chitin, a major component of the shells of crustacea such as crab, shrimp, and crawfish, has been applied in the fields of medicine, food, chemical engineering, pharmaceuticals, nutrition, environmental protection and agriculture [12,13]. In particular, chitosan not only has several advantages over other types of bactericides [14], but also has strong antibacterial activity against a variety of bacteria [15,16,17,18,19,20]. However, unfortunately, in our previous studies, chitosan solution possessed a limited antibacterial activity against Bcc bacteria [4,11].

Interestingly, previous studies have revealed that the biological activities of chitosan and its derivatives could be affected by different environments [14,21] and improved by either combining chitosan with different metal ions [22,23,24,25] or cross-linking chitosan with other organic compounds [26,27,28]. Indeed, chitosan has been cross-linked with glutaraldehyde in several studies [27,29], while these cross-linked complexes have been found to play a key role in the uptake of heavy metals [30,31]. However, to the best of our knowledge, little is known about the antibacterial activity of these cross-linked complexes against Bcc bacteria.

The aim of this study was to synthesize and characterize a cross-linked complex of chitosan and glutaraldehyde with strong anti-Bcc activity.

## 2. Results and Discussion

Results from this study indicated that bacterial growth was unaffected by solid chitosan, but was strongly inhibited by the solid cross-linked chitosan-glutaraldehyde (CLCG) with cross-linking degree of 80.8% regardless of bacterial species and incubation time. In addition, high temperature treated CLCG showed strong antibacterial activity against the selected strain 0901 of the Bcc although the inhibitory effects varied with different temperatures. The differential antibacterial activity between CLCG and chitosan may be mainly due to the difference in their physical-chemical properties, which were evidenced by scanning electron microscopic (SEM) observation, Fourier transform infrared (FTIR) spectra, X-ray diffraction (XRD) pattern, as well as elemental and thermo gravimetric analysis. To the best of our knowledge, this study first synthesized the glutaraldehyde cross-linked chitosan with strong anti-Bcc activity.

### 2.1. Antibacterial Activity of CLCG

This study showed that no inhibition zones were observed when the nine Bcc strains were grown in the presence of solid chitosan in LB medium after 12, 24 and 48 h of incubation, indicating that solid chitosan used in this study has no antibacterial activity against these Bcc strains regardless of incubation time (Table 1). The non-inhibitory effect of chitosan may be due to the fact that chitosan is incapable to diffuse through the adjacent agar media when it is in a solid form [32]. Furthermore, this result is consistent with a number of previous studies that demonstrated chitosan particles would exhibit their potential in suppressing the bacterial growth only when they are in acidic media, in which the NH_2_ group in chitosan becomes a quaternary amino group and allows the chitosan to inhibit the growth of a variety of bacteria [11,33,34].

**Table 1:** Antibacterial activity of chitosan and cross-linked chitosan-glutaraldehyde (CLCG) against *Burkholderia cepacia* complex after different incubation time.

Treatments	Inhibition diameter (mm) after incubation of		
	12 h	24 h	48 h
*B. multivorans* PW99			
Chitosan	0.0 ± 0.0 a	0.0 ± 0.0 a	0.0 ± 0.0 a
CLCG	20.0 ± 2.1 d	19.8 ± 2.1 d	19.6 ± 3.2 d
*B. stabilis* M8			
Chitosan	0.0 ± 0.0 a	0.0 ± 0.0 a	0.0 ± 0.0 a
CLCG	17.6 ± 1.5 c	17.5 ± 1.5 c	17.3 ± 1.5 c
*B. seminalis* R456			
Chitosan	0.0 ± 0.0 a	0.0 ± 0.0 a	0.0 ± 0.0 a
CLCG	15.5 ± 1.5 b	15.3 ± 1.4 b	15.0 ± 1.6 b
*B. seminalis* S9			
Chitosan	0.0 ± 0.0 a	0.0 ± 0.0 a	0.0 ± 0.0 a
CLCG	16.5 ± 1.6 bc	16.3 ± 1.6 bc	16.1 ± 2.0 bc
*B. vietnamiensis* S23			
Chitosan	0.0 ± 0.0 a	0.0 ± 0.0 a	0.0 ± 0.0 a
CLCG	16.6 ± 0.7 bc	16.4 ± 0.9 bc	16.1 ± 0.8 bc
*B. contaminans* Y4			
Chitosan	0.0 ± 0.0 a	0.0 ± 0.0 a	0.0 ± 0.0 a
CLCG	24.0 ± 1.2 g	23.5 ± 1.4 f	23.4 ± 1.6 f
*B. cenocepacia* Y8			
Chitosan	0.0 ± 0.0 a	0.0 ± 0.0 a	0.0 ± 0.0 a
CLCG	22.6 ± 2.0 f	22.3 ± 2.0 ef	22.1 ± 2.2 e
*B. cenocepacia* Y17			
Chitosan	0.0 ± 0.0 a	0.0 ± 0.0 a	0.0 ± 0.0 a
CLCG	20.3 ± 1.6 de	20.0 ± 1.5 d	19.9 ± 1.7 d
*B. seminalis* 0901			
Chitosan	0.0 ± 0.0 a	0.0 ± 0.0 a	0.0 ± 0.0 a
CLCG	21.3 ± 2.7 e	21.0 ± 2.9 de	20.9 ± 2.9 de

Data were pooled from two independent experiments and shown as means ± standard error. Means in a column followed by the same letter are not significantly different (*p* < 0.05).

In contrast with the result of chitosan, clear inhibitory zones were produced by CLCG, while the inhibition zone diameter of CLCG against the nine Bcc strains is significantly greater than the corresponding that of chitosan after 12, 24 and 48 h of incubation (Table 1). This result revealed that CLCG markedly suppressed the growth of the nine Bcc strains regardless of bacterial species and incubation time. Furthermore, this result in this study is generally consistent with the result of several previous studies, which showed that crosslinking by glutaric dialdehyde might aggravate the inhibitory effect of chitosan against some bacteria such as *Escherichia coli* and *Bacillus subtilis* [35,36]. In addition, Mcconnell * et al.* [37] reported that noncrosslinked chitosan films were digested by both pancreatic and colonic enzymes produced by the human colonic bacteria, while glutaraldehyde crosslinked chitosan films were resistant to both enzyme systems. Therefore, it could be suggested that CLCG might be able to be used as a promising novel marine antibacterial agent.

In general, the diameter of the inhibition zone for CLCG against the nine Bcc strains was very slightly reduced with the increase of incubation time, indicating that the antibacterial activity of CLCG was unaffected by incubation time. In contrast, the difference in inhibition zone diameter was noted between Bcc species. Indeed, the maximum inhibition zone diameter of 24.0, 23.5 and 23.4 mm was reached by *B. contaminans* strain Y4, while the minimum inhibition zone diameter of 15.5, 15.3 and 15.0 mm was obtained by *B. seminalis* strain R456 after 12, 24 and 48 h of incubation, respectively (Table 1). This result revealed the differential sensitivity of Bcc species to CLCG. However, compared to chitosan, CLCG showed stronger antibacterial activity against the nine Bcc strains regardless of bacterial species (Table 1).

In addition to the difference in CLCG sensitivity among the Bcc species, strains within a Bcc species also exhibited differential sensitivity to CLCG. Indeed, there was no significant difference in the diameter of the inhibition zone between strain R456 and S9 of *B. seminalis* regardless of incubation time. In contrast, the diameter of the inhibition zone of *B. cenocepacia* strain Y8 is significantly greater than that of *B. cenocepacia* strain Y17 regardless of incubation time (Table 1). The difference in the sensitivity of Bcc strains to CLCG may be attributed to the complex interaction between CLCG and Bcc bacteria. However, the growth of all tested Bcc strains was significantly inhibited by CLCG compared to chitosan (Table 1).

**Figure 1:**
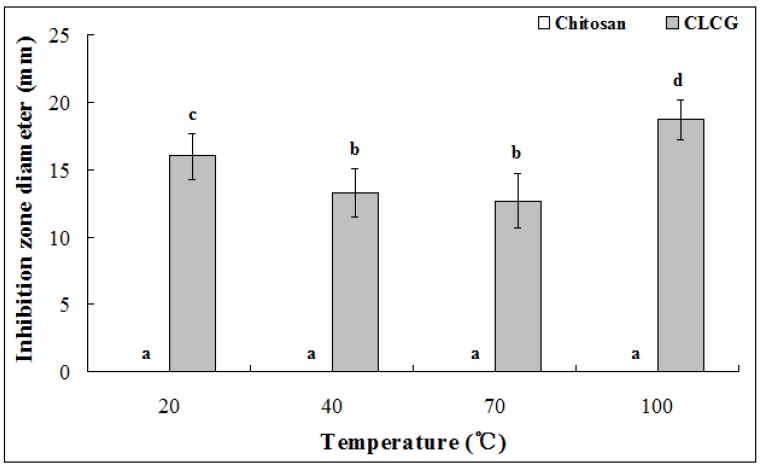
Effect of temperature on antibacterial activity of chitosan and cross-linked chitosan-glutaraldehyde (CLCG) against *Burkholderia seminalis* strain 0901. Data from the repeated experiment were pooled and subjected to analysis of variance. Columns with the same letters are not significantly different (*p* < 0.05). Error bars represent the standard error of the mean.

This study also indicated that no inhibition zone was observed when the selected strain 0901 of the Bcc was grown in the presence of heat treated chitosan in LB medium, indicating that solid chitosan has no antibacterial activity against Bcc strain 0901 even if after different heat treatment (Figure 1). In contrast, CLCG treated at 20 °C, 40 °C and 70 °C produced clear inhibitory zones. Indeed, the inhibition zone diameter of CLCG that treated at 20 °C for 2 h was 16.5 mm. When CLCG was treated at 40 °C and 70 °C for 2 h, the inhibition zone diameter was significantly reduced by 30.4% and 35.6%, respectively, compared to that of CLCG treated at 20 °C. When heat treated temperature was 100 °C, the inhibition zone diameter of CLCG was increased by 45.6% compared to that of CLCG treated at 20 °C (Figure 1). Although the antibacterial activity of CLCG against Bcc strain 0901 would show different when CLCG was treated at different temperature, this result clearly indicated that heat treated CLCG had stronger antibacterial activity against Bcc strain 0901 compared to the corresponding heat treated chitosan regardless of the heat treated temperature.

The result from this study also revealed that the antibacterial activity of CLCG may be mainly from itself, but not from either chitosan or glutaraldehyde. Indeed, the effect of chitosan could be easily excluded because solid chitosan used in this study shows no antibacterial activity against the Bcc strains. On the other hand, it is well known that 2% alkaline glutaraldehyde is the most widely used disinfectant. However, the risk of glutaraldehyde contamination has been markedly reduced by removing the non cross-linked glutaraldehyde from air dry CLCG sample with double distilled water. Furthermore, the weak acid chitosan solution in this study may abolish the antibacterial effect of glutaraldehyde for the glutaraldehyde activity at low concentrations is favored by an alkaline pH. In addition, the diameter of the inhibition zone for CLCG against Bcc strain 0901 was significantly affected by heat treatment, indicating that the antibacterial activity of CLCG mainly depends on the crosslink reaction between chitosan and glutaraldehyde.

The interaction between chitosan with glutaraldehyde have received considerable attention over the past few decades [35,38], while the obtained data from ^13^C NMR, infrared and Raman spectroscopies evidenced the formation of an ethylenic double bond in the chitosan–glutaraldehyde interaction [39]. In addition, two main crosslinking mechanisms, involving formation of Schiff’s base structures or Michael-type adducts, have been proposed for the reaction of chitosan and glutaraldehyde. However, the nature of the chemical bonding between chitosan amine groups and glutaraldehyde has been subject of controversy, which may be due to this reason that the rate of chemical gelation of chitosan chains with glutaraldehyde depends on various parameters, namely pH, ionic strength, temperature, chitosan concentration and degree of crosslinking [38,40].

This result indicated that the inhibition in bacterial growth should be mainly due to the direct *in vitro* antibacterial activity of CLCG. However, Zhang *et al.* [35] found that in addition to increased antibacterial activity, glutaric dialdehyde crosslinking also enhanced chitosan uptake on the surface of cotton fabrics, with good durability of antibacterial properties to washing. This may be due to that glutaric dialdehyde is a binary aldehyde compound. One aldehyde group of glutaraldehyde reacts with amino group of chitosan, contributing to the antibacterial activity, while the other reacts with cellulose of cotton fabrics, improving the fastness property. Therefore, it could be expected that crosslinking by glutaraldehyde may have a direct and indirect contribution to the antibacterial activity of CLCG.

### 2.2. Cross-Linking Degree of CLCG

Results from this study indicated that the cross-linking degree of the synthesized CLCG with strong anti-Bcc activity was 80.8%, while the other synthesized products with different cross-linking degrees were discarded for limited anti-Bcc activities (data not shown). This result is in agreement with the result of previous studies [41,42,43], which reported that in addition to degree of deacetylation in chitosan, the degree of crosslinking also controls the properties of chitosan. In addition, a number of studies have revealed that the aldehyde groups react immediately with the –NH_2_ groups along the chitosan chains after introducing wet chitosan microspheres into glutaraldehyde solution [29,35,38,42,44,45]. Therefore, it could be suggested that CLCG might be formed by cross-linking chitosan with glutaraldehyde at amino groups.

### 2.3. SEM of CLCG

**Figure 2:**
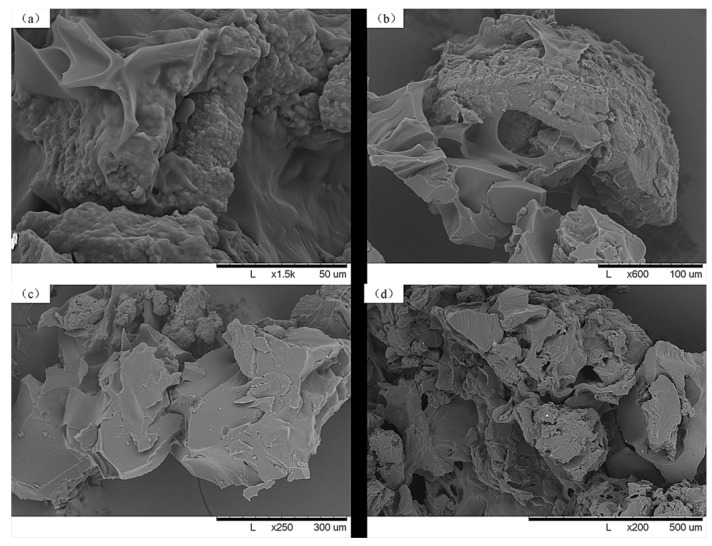
SEM images of cross-linked chitosan-glutaraldehyde (CLCG) at four different magnifications (**a**) 1500×; (**b**) 500×; (**c**) 250×; and (**d**) 200×.

Figure 2 presents the SEM micrographs illustrating the surface morphology of CLCG at four different (1500×, 500×, 250× and 200×) magnifications. In general, this result clearly indicated that there is a difference in the surface microscopic morphology between chitosan and CLCG. Indeed, compared to the smooth, dense and flat morphology of chitosan [46], the SEM images in this study revealed that CLCG had a rough and porous surface (Figure 2), which is consistent with the result of previous studies [29,35,47]. In contrast, Wang * et al.* [48] and Kulkarni *et al.* [49] revealed that the microspheres of glutaraldehyde-crosslinked chitosan were spherical and have a smooth outer structure. Furthermore, Fang and Hu [50] found that the ultrastructure of the glutaraldehyde-crosslinked chitosan is a uniform and cloud-like. These differential results should be attributed to the fact that cross-linking degree and degree of deacetylation influences the size and morphology of glutaraldehyde-crosslinked chitosan [47,51].

The rough surface morphology may be due to the insufficient cross-linking of CLCG or the glutaraldehyde groups partly grafted on chitosan, indicating that the reaction has taken place on the surface. Furthermore, the porous structure of CLCG in this study may offer more adsorption sites for adsorbate, which generally supported the fact that glutaraldehyde-crosslinked chitosan has been widely applied in the uptake of heavy metals [30,31] and drug delivery [51]. In addition, CLCG with a higher total surface area and a more open pore structure could be supposed to be favorable to interact with the Bcc strains, which may at least partially explain this result that the growth of the Bcc strains were significantly inhibited by CLCG, but not solid chitosan.

### 2.4. FITR Spectrum of CLCG

**Figure 3:**
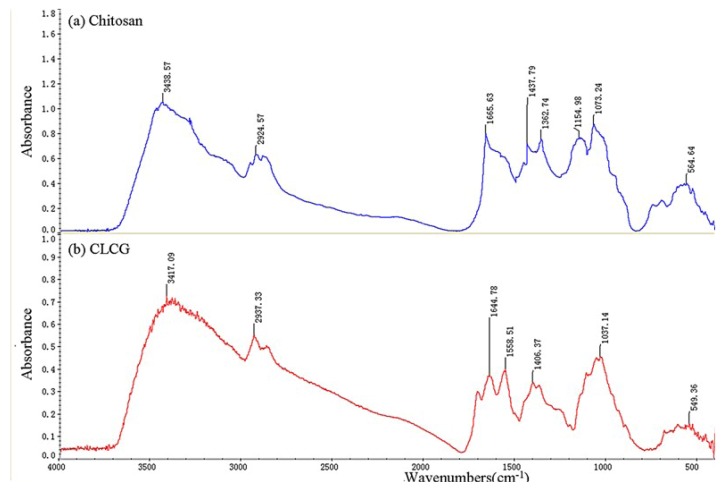
FTIR spectra of (**a**) chitosan and (**b**) cross-linked chitosan-glutaraldehyde (CLCG).

FTIR spectra of chitosan and CLCG were displayed in Figure 3a,b, respectively. The main peaks for chitosan can be assigned as follows: 3439 cm^−1^ (N–H and O–H stretching vibration), 2925 cm^−1^ (CH_3_ symmetric stretch), 1666 cm^−1^ (C=O stretching vibration), 1438 cm^−1^ (C–N stretching vibration), 1363 cm^−1^ (CH_3_ bending vibration), 1155 cm^−1^ (C–O–C bending vibration), and 1073 cm^−1^ (C–OH stretching vibration). However, some major changes have been observed in the spectrum of CLCG by comparing the spectral differences in the 4000–500 cm^−1^ region of FTIR spectra between chitosan and CLCG. The FTIR spectrum of CLCG revealed that the N–H and O–H stretching vibration at 3439 cm^−1^ shifts to 3417 cm^−1^, the CH_3_ symmetric stretch at 2925 cm^−1^ shifts to 2937 cm^−1^, the C=O stretching vibration at 1666 cm^−1^ shifts to 1645 cm^−1^, the C–N stretching vibration at 1438 cm^−1^ shifts to 1406 cm^−1^, and the C–OH stretching vibration at 1073 cm^−1^ shifts to 1037 cm^−1^ (Figure 3). Furthermore, the CH_3_ bending vibration at 1363 cm^−1^ and C–O–C bending vibration at 1155 cm^−1^ were observed in FITR of chitosan, but not in FITR of CLCG. In contrast, the band at 1559 cm^−1^ (amide II) was found in FITR of CLCG, but not in FITR of chitosan (Figure 3).

The result from this study is consistent with many previous studies [26,29,52], which have demonstrated that there was a difference in FITR profile between chitosan and CLCG. The peaks of chitosan at 1363 cm^−1^ and 1155 cm^−1^ disappeared, indicating that the two peaks may be hindered by glutaraldehyde cross-linked structure of chitosan. The appearance of the peak at 1559 cm^−^^1^ can be attributed to the crosslinking reaction of chitosan and glutaraldehyde. Indeed, Ramachandran *et al.* [53] found that the new sharp peak at 1610 cm^−1^ represents stretching vibrations of C=N in Schiff’s base formed by the reaction of glutaraldehyde and chitosan. Furthermore, Knaul *et al.* [45] found that the chitosan film that reacted with glutaraldehyde exhibits a strong absorbance at 1664 cm^−1^, while Gupta and Jabrail [41] reported that the IR spectra have shown a strong absorption band at 1660 cm^−1^ in the glutaraldehyde cross-linked chitosan microsphere. In addition, Oyrton *et al.* [39] revealed that the increase of glutaraldehyde in the sequence of these modified chitosans caused a successive increase in intensity of ethylenic bond frequency at 1562 cm^−1^.

The result from this study indicated that the amide II band at 1559 cm^−^^1^ that may contribute to the amine–NH_2_ group was specific for CLCG. Interestingly, Tripathi *et al.* [32] revealed the change in the characteristic shape of the chitosan spectrum after cross-linking while stretching vibration spectra of the amide group of chitosan-based antimicrobial films appear at 1560 cm^−^^1^. Furthermore, Zhang *et al.* [35] reported that the intensity at 1557.19 reveals the quaternary-amino band. Thus, the antibacterial activity of CLCG may be due to the positive charge NH_2_^+^, which was expected to interact with gram-negative bacterial surface that predominantly consisted of anionic components, such as lipopolysaccharides, phospholipids, and lipoproteins. Indeed, previous studies have indicated that this charge interaction could disrupt the organization of the outer membrane in bacteria and increase its permeability [11,14]. Therefore, it could be suggested that the inhibitory activity of CLCG to the Bcc strains is mainly comes from its positive charge.

### 2.5. X-ray Diffraction Analysis

The change of chitosan structure before and after cross-linking with glutaraldehyde was investigated by means of powder XRD, which is a proven tool to study crystal lattice arrangements and yields very useful information on degree of sample crystallinity. The XRD pattern of chitosan in this study displayed two sharp diffraction peaks at 2*θ* = 10° and 20°, revealing the high crystallinity of chitosan. This result is consistent with the result of a number of previous studies [22,32,33], which reported the typical X-ray diffraction pattern of chitosan. The high crystallinity is due to the chitosan structure while plenty of hydroxyl and amino groups could form strong intermolecular and intramolecular hydrogen bonds [33]. In addition, the structure of chitosan molecules has certain regularity. As a result, chitosan molecules could form crystalline regions very easily.

**Figure 4:**
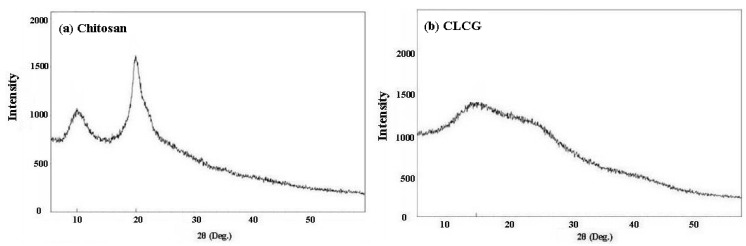
X-ray diffractograms of (**a**) chitosan and (**b**) cross-linked chitosan-glutaraldehyde (CLCG).

However, as regards CLCG, the characteristic peaks at 2*θ* = 10° and 20° disappeared, and a very weak and broad peak centered at 2*θ* = 15° appeared (Figure 4). This difference in XRD patterns between chitosan and CLCG should be attributed to the cross-linking reaction between chitosan and glutaraldehyde. In agreement with the result of previous studies [32,33,39], the crystallinity of CLCG decreases after crosslinking with glutaraldehyde. This could be attributed to the deformation of the strong hydrogen bond in original chitosan due to the substitution of hydroxyl and amino groups, which efficiently destroyed the regularity of the packing of the original chitosan chains and resulted in the formation of amorphous CLCG.

Recently, Mohamed and Fahmy [54] revealed that the incorporation of hydrophilic cross-linker into chitosan allowed the synthesis of hydrogels with higher hydrophilicity, with greater positive charge density and with higher antimicrobial activities, which may explain the result that CLCG had a better antibacterial activity against the Bcc strains compared to the solid chitosan. As mentioned above, the antibacterial mechanism of CLCG may be mainly attributed to the interaction between positive charged CLCG molecules and negatively charged bacterial cell membranes. It suggests that the greater the number of cationized groups, the higher the antibacterial activity. Furthermore, the decrease in intermolecular hydrogen bonds of CLCG could result in the increase of its solubility, which is able to facilitate the penetration of CLCG into the cells of bacteria, thereby preventing the transformation of DNA to RNA to obtain a higher antibacterial activity. In addition, the antibacterial activity of the amorphous CLCG against the Bcc strains may partially due to its ability to chelate metal ions that bind to nutrients essential to bacterial growth.

### 2.6. Elemental Analysis of CLCG

Results from this study indicated that there was a difference in the elements between chitosan and CLCG. Indeed, the element analysis indicated that CLCG had a composition of 33.5% (w/w) carbon, 6.5% (w/w) hydrogen and 1.6% (w/w) nitrogen. In agreement with the result of Gupta *et al.* [41], this study indicated that the contents of carbon, hydrogen and nitrogen in CLCG were lower than the corresponding contents in chitosan, which reduced by 17.2%, 7.9% and 77.4%, respectively (Table 2). In contrast, Oyrton *et al.* [39] found that the content of carbon and hydrogen percentages is followed by an increase of glutaraldehyde in the chitosan sequence of modified polymers, while the amount of nitrogen decreases with the increasing degree of glutaraldehyde in this series of samples. Therefore, this differential result may be attributed to the difference in the degree of crosslinking.

**Table 2:** Elementary analysis of chitosan and cross-linked chitosan-glutaraldehyde (CLCG).

Substance	Element (%, w/w)		
	Carbon	Hydrogen	Nitrogen
Chitosan	40.47	7.08	7.21
CLCG	33.50	6.52	1.63

From this result, it could be supposed that the cross-linking reaction happened and new functional groups were produced, while the antibacterial activity of CLCG against the Bcc strains is associated with the decrease in the contents of carbon, hydrogen and nitrogen. Indeed, the reduction in the elements could be mainly attributed to the cross-linking reaction between the aldehyde groups of glutaraldehyde and amino groups of chitosan, which resulted in the formation of amorphous CLCG with higher hydrophilicity. Furthermore, as a matter of fact, the presence of H and O from moisture is also associated to the decrease in contents of the elements, in particular, carbon, and nitrogen. In addition, the loosely packed structure has obviously played an important role in the antibacterial activity of CLCG against the Bcc strains, which has been described as above.

### 2.7. Thermo Gravimetric Analysis of CLCG

**Figure 5:**
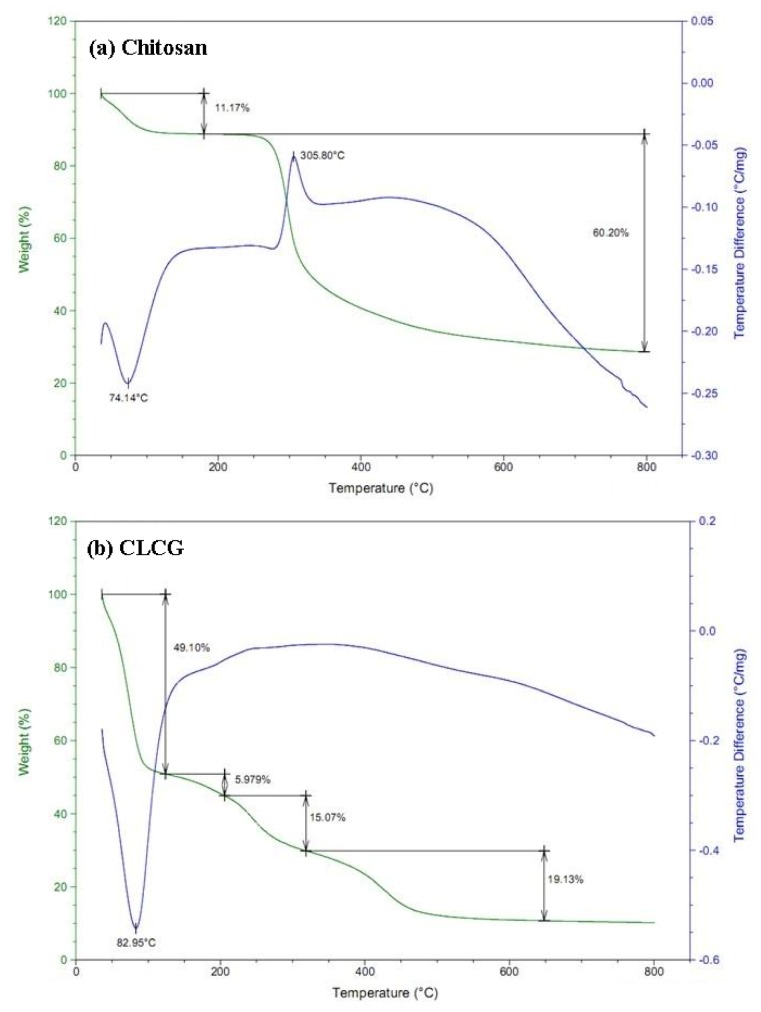
Thermo gravimetric analysis of (**a**) chitosan and (**b**) cross-linked chitosan-glutaraldehyde (CLCG).

Thermo gravimetric analysis for the experimental samples of chitosan and CLCG were illustrated in Figure 5 and the corresponding thermal degradation values were displayed in Table 3. This result revealed that the thermal degradation curves of chitosan and CLCG were dependent on the temperature, which is consistent with the above result that the antibacterial activity of CLCG was slightly changed when it was treated under different temperature. In general, this result revealed that the thermal stability of CLCG is lower than that of chitosan regardless of the temperature. The weight loss of CLCG is 368.1%–384.7% higher than that of chitosan in the first stage (100 °C–200 °C), 96.8%–384.7% higher than that of chitosan in the second stage (200 °C–300 °C) and 25.7%–96.8% higher than that of chitosan in the third stage (300 °C–800 °C) of thermal degradation.

**Table 3:** Thermal analysis of chitosan and cross-linked chitosan-glutaraldehyde (CLCG) under N_2_ air atmosphere.

Temperature (°C)	% of weight loss (heating rate 20 °C/min)	
	Chitosan	CLCG
100	10.13	47.42
200	11.25	54.53
300	35.06	68.99
400	59.27	76.59
500	60.08	87.80
600	68.39	89.07
700	70.21	89.50
800	71.43	89.81

Previous studies have indicated that the weight loss in the first stage may be mainly attributed to water loss, while in the second and third stage may be mainly due to the breakage of main chain and decomposition of glucose units, respectively [32,55]. Interestingly, results from this study indicated that the weight loss of CLCG at 800 °C is 30.2% higher than that of CLCG at 300 °C, while the weight loss of chitosan at 800 °C is 103.7% higher than that of chitosan at 300 °C, revealing that CLCG is more stable in the glucose units under high temperature compared to chitosan. Therefore, it could be suggested that the weight loss of CLCG may be mainly due to the loss of water.

The result from this study indicated that CLCG has a high water content, while solid chitosan used in this study was unable to be dissolved in water. This revealed that the antibacterial activity of CLCG may be due to its high hydrophilicity. Furthermore, Zhao *et al.* [55] found that the drop in the temperature of water loss and the increase in the moisture content were related to the increase in pore spaces resulting from crosslinking. Therefore, the increased antibacterial activity of CLCG was able to be attributed to the difference in the thermal degradation curve between chitosan and CLCG. In addition, this result of the thermal degradation curve also indicated that new functional groups maybe have been produced due to the cross-linking reaction, which is consistent with the results of SEM, FTIR spectra, XRD pattern and elemental analysis.

## 3. Experimental Section

### 3.1. Chitosan, Glutaraldehyde and Bacteria

Chitosan (molecular weight of 1129 KDa, degree of *N*-deacetylation no less than 85%, practical grade, from crab shells) was obtained from Sigma-Aldrich (St. Louis, MO, USA), while glutaraldehyde was purchased as a 25% stock from Sigma Chemical Co. (St. Louis, MO, USA). In addition, the tested Bcc strains were shown in Table 1, which have been isolated in our previous studies and deposited in the culture collection of the Institute of Biotechnology, Zhejiang University, China.

### 3.2. Cross-Linking of Chitosan and Glutaraldehyde

CLCG was synthesized by dissolving 2.0 g of chitosan into 50.0 mL of 5.0% acetic acid and then adding 15.0 mL 25% glutaraldehyde into this chitosan solution to form a water gel after 24 h of stirring at room temperature by using a magnetic stirrer, while the non cross-linked glutaraldehyde was removed by washing the cross-linked complex more than eight times with double distilled water. The obtained CLCG was then lyophilised and stored at −70 °C.

### 3.3. Antibacterial Activity

The antibacterial activity of CLCG at room temperature (20 °C) against nine different Bcc strains was performed in LB medium according to the method of stainless steel cylinders as described by Li *et al.* [56]. In brief, the final concentration of the Bcc strains in LB medium was 10^8^ CFU/mL, while each stainless steel cylinder was covered with a thin layer of CLCG (about 0.05 g). Each treatment was replicated using five stainless steel cylinders, and the experiment was performed three times.

The effect of temperature in the antibacterial activity of CLCG was carried out by incubating CLCG in sterile water of 40 °C, 70 °C and 100 °C for 2 h, respectively, and then washing CLCG twice with distilled water. In addition, the effect of time in the antibacterial activity of CLCG was performed by incubating CLCG and bacteria for 12 h, 24 h, and 48 h, respectively. The antibacterial activity of CLCG under different environments was determined as described above.

### 3.4. Determination of Cross-Linking Degree

The cross-linking degree of CLCG is representative of a ratio of mass of cross-linked state of CLCG to the whole mass of CLCG. The cross-linking degree of the obtained CLCG was determined by dissolving CLCG into 2.0% acetic acid for 24 h and then measuring the dry weight of pre and post-dissolved CLCG as described by Mitra *et al.* [26].

### 3.5. Scan Electron Microscope

The CLCG sample was washed twice with 0.1 M phosphate buffer solution (pH 7.0, PBS) and fixed with 2.5% (v/v) glutaraldehyde in 0.1 M PBS. Then the CLCG sample was postfixed with 1% (w/v) OsO4 in 0.1 M PBS for 1 h at room temperature and washed three times with the same buffer, dehydrated separately at 4 °C for 15 min in a graded series of ethanol solutions (70, 80, 90, 95 and 100%, v/v), then embedded in Epon 812 a low-viscosity embedding medium. The thin section specimen was cut with a diamond knife on an Ultracut Ultramicrotome (Super Nova; Reichert-Jung Optische Werke, Wien, Austria) and double-stained with saturated uranyl acetate and lead citrate. As described by Wang *et al.* [57], the grids were examined by using a JEM-1230 transmission electron microscope (Hitachi, Tokyo, Japan) at an operating voltage of 75 kV.

### 3.6. Fourier Transform Infrared Spectra

The samples of chitosan and CLCG were first grounded into fine particles using mortar and pestle. The 1.0–2.0 mg of each sample was then mixed with 200 mg potassium bromide (KBr) which extensively dried in microfuge tubes using a lyophiliser. The mixtures have been dried for an additional 2 h in the same microfuge tubes. The KBr based pellets were then compressed into a thin disk by establishing pressure of (5–10) × 10^7^ Pa. The ATR measurements were performed using an FTIR spectrometer (VERTEX 70).

Pellets were scanned at 4 cm^−1^ resolution with 100 scans in the spectral range of 4000–500 cm^−1^ at room temperature. The sample compartment in the FTIR spectrometer was continuously purged with dry air to prevent water vapor. Analysis of the spectral data was performed as described by Garip *et al.* [58] by using Grams 32 (Galactic Industries, Salem, NH, USA) software. The spectral range of 4000–500 cm^−1^ was analyzed. The band positions were measured according to the center of weight and the spectra obtained from the same experimental groups, baseline correction, normalization and the band areas were averaged. The average spectra and normalization process were applied only for visual representation of the differences between chitosan and CLCG, while each original baseline corrected spectrum was taken into consideration for the determination of the spectral parameters and calculation of mean values.

### 3.7. X-ray Diffraction Analysis

The XRD measurements of chitosan and CLCG were recorded as described by Ge *et al.* [22] and Zhang *et al.* [59] by using an XPert PRO diffractometer (Holland) with a detector operating under a voltage of 40.0 kV and a current of 30.0 mA using CuKo radiation. The recording range of 2*θ* was 5° to 60°, and the scanning speed was 6°/min.

### 3.8. Elemental Analysis

The contents of carbon, hydrogen and nitrogen elements in chitosan and CLCG were determined according to the method of Yao *et al.* [52], which were performed on a vario microorganic elemental analyzer (CE Intrusment EA1112, Italy).

### 3.9. Thermo Gravimetric Analysis

Thermal decomposition analysis of chitosan and CLCG was carried out as described by Mitra *et al.* [26] under nitrogen flow (40 & 60 mL/min) with ramp 10 °C/min using Universal V4.3A TA instruments. The temperature range is between 0 °C and 800 °C.

### 3.10. Statics Analysis

The software STATGRAPHICS Plus, version 4.0 (Copyright Manugistics Inc., Rockville, MD, USA) was used to perform the statistical analysis. Levels of significance (*p* < 0.05) of main treatments and their interactions were calculated by analysis of variance after testing for normality and variance homogeneity.

## 4. Conclusions

Results from this study indicated that the growth of Bcc strains was unaffected by solid chitosan, but was strongly inhibited by the synthesized glutaraldehyde cross-linked chitosan with cross-linking degree of 80.8% regardless of bacterial species and incubation time. Furthermore, CLCG treated with high temperature showed antibacterial activity against the selected strain 0901 of the Bcc although the inhibitory effects varied with different temperatures. In addition, the differential anti-Bcc activity between chitosan and glutaraldehyde cross-linked chitosan may be mainly due to the difference in their physical-chemical properties, which has been determined based on SEM observation, FTIR spectra, X-ray diffraction pattern, as well as elemental and thermo gravimetric analysis. Overall, this study revealed that the use of glutaraldehyde cross-linked chitosan as a potential antibacterial agent seems to be promising in reducing the risk of CF patients.
